# Diagnostic Accuracy of ^18^F-PSMA-1007 PET/CT Imaging for Lymph Node Staging of Prostate Carcinoma in Primary and Biochemical Recurrence

**DOI:** 10.2967/jnumed.120.246363

**Published:** 2021-02

**Authors:** Katharina Sprute, Vasko Kramer, Stefan A. Koerber, Manuel Meneses, Rene Fernandez, Cristian Soza-Ried, Mathias Eiber, Wolfgang A. Weber, Isabel Rauscher, Kambiz Rahbar, Michael Schaefers, Tadashi Watabe, Motohide Uemura, Sadahiro Naka, Norio Nonomura, Jun Hatazawa, Constantin Schwab, Viktoria Schütz, Markus Hohenfellner, Tim Holland-Letz, Juergen Debus, Clemens Kratochwil, Horacio Amaral, Pete L. Choyke, Uwe Haberkorn, Camilo Sandoval, Frederik L. Giesel

**Affiliations:** 1 Department of Nuclear Medicine, University Hospital, Heidelberg, Germany; 2 Positronpharma SA, Santiago, Chile; 3 Center of Nuclear Medicine, PositronMed, Santiago, Chile; 4 Department of Radiation Oncology, Heidelberg University Hospital, Heidelberg, Germany; 5 National Center for Tumor Disease, Heidelberg, Germany; 6 Heidelberg Institute of Radiation Oncology, Heidelberg, Germany; 7 Fundación Arturo Lopez Perez, Santiago, Chile; 8 Department of Nuclear Medicine, Munich University Hospital, Munich, Germany; 9 Department of Nuclear Medicine, Muenster University Hospital, Muenster, Germany; 10 Department of Nuclear Medicine and Tracer Kinetics, Osaka University Graduate School of Medicine, Osaka, Japan; 11 Department of Urology, Osaka University Graduate School of Medicine, Osaka, Japan; 12 Osaka University Hospital, Osaka, Japan; 13 Research Center for Nuclear Physics, Osaka University, Osaka, Japan; 14 Department of Pathology, Heidelberg University Hospital, Heidelberg, Germany; 15 Department of Urology, Heidelberg University Hospital, Heidelberg, Germany; 16 Department of Biostatistics, German Cancer Research Center, Heidelberg, Germany; 17 German Cancer Consortium, Heidelberg, Germany; 18 Department of Radiation Oncology, Heidelberg Ion-Beam Therapy Center, Heidelberg University Hospital, Heidelberg, Germany; 19 Clinical Cooperation Unit Radiation Oncology, German Cancer Research Center, Heidelberg, Germany; 20 Clinical Cooperation Unit Nuclear Medicine, German Cancer Research Center, Heidelberg, Germany; and; 21 Molecular Imaging Program, Center for Cancer Research, National Cancer Institute, National Institutes of Health, Bethesda, Maryland

**Keywords:** ^18^F-PSMA-1007 PET/CT, prostate cancer, histopathology, correlation, lymph node staging

## Abstract

Prostate-specific membrane antigen (PSMA)–ligand PET/CT is performed on patients with prostate cancer to stage the disease initially or to identify sites of recurrence after definitive therapy. On the basis of clinical results, ^18^F-PSMA-1007 is a promising PSMA PET tracer, but detailed histologic confirmation has been lacking. **Methods:** Ninety-six patients with prostate cancer underwent ^18^F-PSMA-1007 PET/CT followed by either radical prostatectomy with lymphadenectomy or salvage lymphadenectomy. The histologic findings of PSMA PET–positive nodes were analyzed retrospectively. A lesion-based and patient-based analysis was performed comparing all positive lesions and only lesions larger than 3 mm on histopathology. **Results:** Of the patients, 90.6% received ^18^F-PSMA-1007 PET/CT for staging before the primary treatment, whereas 9.4% underwent imaging for biochemical recurrence. In 34.4% of the cohort, positive lymph nodes were present on imaging. In total, 1,746 lymph nodes were dissected in 96 patients. ^18^F-PSMA-1007 PET had a lesion-based sensitivity of 81.7%, a specificity of 99.6%, a positive predictive value of 92.4%, and a negative predictive value of 98.9% for detecting positive lymph nodes larger than 3 mm. In the analysis of all malignant nodes regardless of size, the overall sensitivity, specificity, positive predictive value, and negative predictive value on lesion-based analysis were 71.2%, 99.5%, 91.3%, and 97.9%, respectively. The patient-based analysis showed a sensitivity of 85.9% and a specificity of 99.5% for lymph nodes larger than 3 mm. **Conclusion:** ^18^F-PSMA-1007 PET/CT reliably detects malignant lymph nodes and has an exceptional specificity of more than 99% for nodal metastases.

Prostate cancer is one of the most commonly diagnosed cancers worldwide, and the number of newly diagnosed patients is increasing commensurate with an aging population (1) Prostate-specific membrane antigen (PSMA)–ligand PET/CT is increasingly used in the initial staging of high-risk tumors and in identifying sites of recurrence. Several tracers have been developed for PSMA PET/CT. A study that compared conventional imaging and ^68^Ga-PSMA-11 PET/CT found PSMA PET/CT to be superior (2). For the most studied tracer, ^68^Ga-PSMA-11, high rates of detection have been reported on the basis of clinical and histologic data (3–5). ^18^F-PSMA-1007 offers a lower urinary clearance and a longer half-life. The decreased activity in the bladder facilitates the detection of local recurrence. In a direct comparison between ^68^Ga-PSMA-11 PET/CT and ^18^F-PSMA-1007 PET/CT, the latter was superior in the detection of lesions close to the bladder and ureter (6). Moreover, ^18^F-PSMA-1007, with its longer-lived radioisotope and higher positron yield, promises advantages in imaging quality and sensitivity (7). However, since its recent introduction, only a few studies with ^18^F-PSMA-1007 have validated this tracer with histopathology. In a recent study involving 8 patients with ^18^F-PSMA-1007 imaging and histopathology, a sensitivity of 94.7% was reported for patients undergoing radical prostatectomy and extended lymphadenectomy (8). A different study of 10 patients with ^18^F-PSMA-1007 reported a sensitivity of 71%, specificity of 81%, positive predictive value (PPV) of 83%, and negative predictive value (NPV) of 68%, with an accuracy of 75% for total agreement (9).

The aim of this retrospective, multicenter study was to determine the diagnostic accuracy of ^18^F-PSMA-1007 PET/CT imaging for N-staging of prostate cancer initially and for the assessment of nodal recurrence using histopathology as the gold standard.

## MATERIALS AND METHODS

### Study Design and Patient Population

From the databases of 5 institutions, we identified patients who underwent ^18^F-PSMA-1007 PET/CT imaging followed by surgery with histopathologic evaluation of surgical specimens. The PET/CT, followed by either radical prostatectomy with extended lymphadenectomy or salvage lymphadenectomy, was performed between July 2016 and November 2019. The mean time between PET/CT and surgery was 47.65 ± 35.87 d (range, 1–165 d).

Patients for whom data were available only for prostate biopsies, prostatectomies without lymphadenectomies, or bone biopsies were not considered in this study. Only patients for whom an imaging report and a histopathology report were available were selected. Finally, 96 patients were included in this retrospective analysis (43 from Fundación Arturo Lopez Perez, 33 from the University of Heidelberg, 15 from the University of Munich, 4 from the University of Muenster, and 1 from Osaka University Hospital). Patient characteristics are summarized in Table 1.

**TABLE 1:** Patient Characteristics

Characteristic	Data
Age at PET (y)	69.5 (48–78)
Indication for PET/CT	
Initial staging	87 (90.6%)
Recurrence	9 (9.4%)
Prostate-specific antigen value (ng/mL)	10.5 (0.1–120)
At time of PET/CT	
Initial staging	11.7 (0.1–120)
Recurrence	1.8 (0.47–4.7)
Gleason score	
≤6	3 (3.3%)
7	55 (60.4%)
8	6 (6.6%)
9	27 (29.7%)
LN stage according to PET/CT	
N0	63 (65.6%)
N1	33 (34.4%)
LN stage according to histopathology (≥3 mm)	
pN0	65 (67.7%)
pN1	31 (32.3%)
Number of resected LNs	1,746
Number of positive LNs on PET	92
Number of positive LNs on histopathology	117
Number of positive LNs on histopathology > 3 mm	104

Qualitative data are numbers and percentages; continuous data are median and range. *n* = 96 except for Gleason score (*n* = 91).

This retrospective study was approved by every local institutional review board, and all subjects gave written informed consent to anonymized evaluation and publication of their data. All reported investigations were conducted in accordance with the Helsinki Declaration and local regulations.

### Radiochemistry

^18^F-PSMA-1007 was produced in compliance with current good-manufacturing-practice guidelines as described preciously (10). Reagent kits, precursor, and reference standard were obtained from ABX. The radiosynthesis was performed as a single-step radiofluorination on a modified nuclear interface ^18^F-FDG synthesis module (TracerLab FX FN analog; GE Healthcare), a Neptis plug synthesis module (ORA), or a Synthera V2 synthesis module (IBA) using 1.6 mg of PSMA-1007 precursor in dimethyl sulfoxide for 10 min at 80°C, followed by purification on 2 stacked solid-phase extraction cartridges (PS-H+, C18_ec_; Macherey-Nagel) and final dilution with phosphate-buffered saline containing ascorbate to obtain ^18^F-PSMA-1007 after sterile filtration as a solution ready for injection. At Osaka University Hospital, ^18^F-PSMA-1007 was produced under optimized conditions on a MPS200 cassette-type synthesizer (Sumitomo). High-performance liquid chromatography and thin-layer chromatography were performed to test the radiochemical and chemical purity; residual solvents were tested using gas chromatography, and the presence of tetrabutylammonium was determined using a thin-layer chromatography spot test. Further quality control (radionuclide purity, appearance, pH, endotoxins, sterility, filter integrity) was performed in compliance with current pharmacopeias.

^18^F-PSMA-1007 was obtained in radiochemical yields of 50% ± 10% after a total synthesis time of 25–62 min. The radiochemical purity was at least 95% (high-performance liquid chromatography and thin-layer chromatography). The PSMA-1007 content was less than 10 μg/mL, and all other radiopharmaceutical specifications were in accordance with current pharmacopeias.

### Imaging Protocol

Patients were injected with 270 MBq, on average, of ^18^F-PSMA-1007, and images were acquired at an average of 90 min (range, 47–169 min) after injection on either a Biograph mCT (Siemens) or a Discovery 710 (GE Healthcare) PET scanner with similar imaging protocols. A detailed description can be found in Supplemental Table 1 (supplemental materials are available at http://jnm.snmjournals.org).

### Image and Histopathologic Analysis

Extended lymph node (LN) dissection was performed for nearly all patients who received surgery as the primary treatment (86/87), and the extent of the LN dissection was described in the pathology report. In cases of biochemical recurrence, the surgery was PSMA-based. In general, dissected regions and findings were categorized as in the left or right iliac external LNs or the left or right iliac internal LNs. The total number of LNs and the number of histologically tumor–positive LNs were described for each region and then correlated with the PET findings.

Interpretation of all imaging data and their histopathologic correlations were done by each individual center. When there was discordance between imaging findings and histopathology, the data were reevaluated by the team at Heidelberg University Hospital, using a consensus reading by 2 board-certified nuclear medicine physicians with more than 10 y of experience in PET imaging diagnostics.

All lesions suspected of representing prostate cancer or affected LNs on imaging were noted, along with the corresponding side and localization. Focal uptake of ^18^F-PSMA-1007 higher than the surrounding background and not associated with physiologic uptake was considered suggestive of malignancy. Typical pitfalls in PSMA-ligand PET imaging (e.g., celiac and other ganglia, fractures, or degenerative changes) were considered (11).

### Statistical Analysis

Data were tabulated using Excel (Microsoft). On the basis of the PET imaging and histopathology results, patients and LNs were defined as true-positive (positive on PET and histopathology), false-positive (positive on PET, negative on histopathology), true-negative (negative on PET and histopathology), or false-negative (negative on PET, positive on histopathology).

A lesion-based and patient-based analysis was performed to calculate sensitivity, specificity, PPV, NPV, and accuracy as follows: sensitivity was defined as true-positives divided by true-positives plus false-negatives. Specificity was defined as true-negatives divided by true-negatives plus false-positives. PPV was defined as true-positives divided by true-positives plus false-positives. NPV was defined as true-negatives divided by true-negatives plus false-negatives. Accuracy was defined as true-positives plus true-negatives divided by the total population.

In one approach, every LN seen on histopathology was counted; in a different approach, only LNs larger than 3 mm were considered, according to the detection rates of the PET/CT.

## RESULTS

### Lesion-Based Analysis

Lymphadenectomy was performed on all 96 patients, yielding 1,746 LNs. Histology was set as the gold standard. Diagnostic imaging findings were compared with histopathology findings to determine the descriptive statistics. In total, 92 positive LNs were detected on ^18^F-PSMA-1007 PET/CT. Detailed findings for individual patients are listed in Supplemental Tables 2 and 3.

**FIGURE 1.**
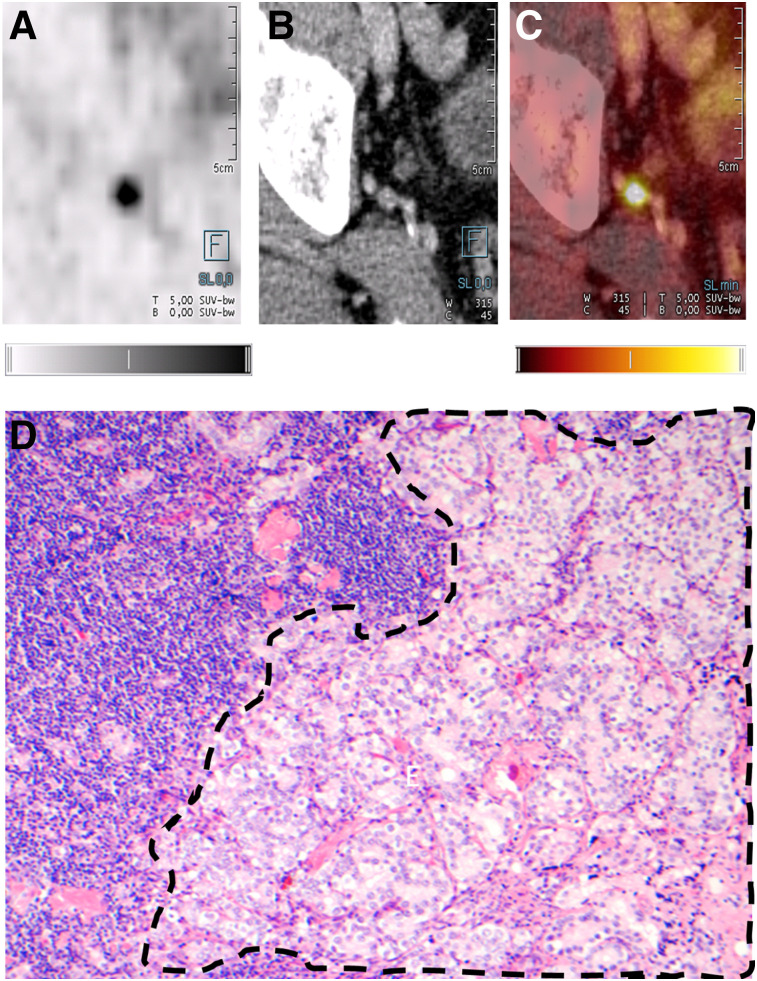
(A–C) Axial ^18^F-PSMA-1007 PET (A), CT (B), and ^18^F-PSMA-1007 PET/CT (C) images of 73-y-old patient with prostate adenocarcinoma (Gleason score, 4 + 3; T3bN1M0; correlation between PET/CT and histopathology for exemplary LN). (D) Confirmed histopathologic staining (hematoxylin and eosin, ×10) of acinar structures of prostate adenocarcinoma (dashed outline) adjacent to normal LN structures. Quantitative gray scale and color scale represent SUV from 0.00 to 5.00.

**FIGURE 2.**
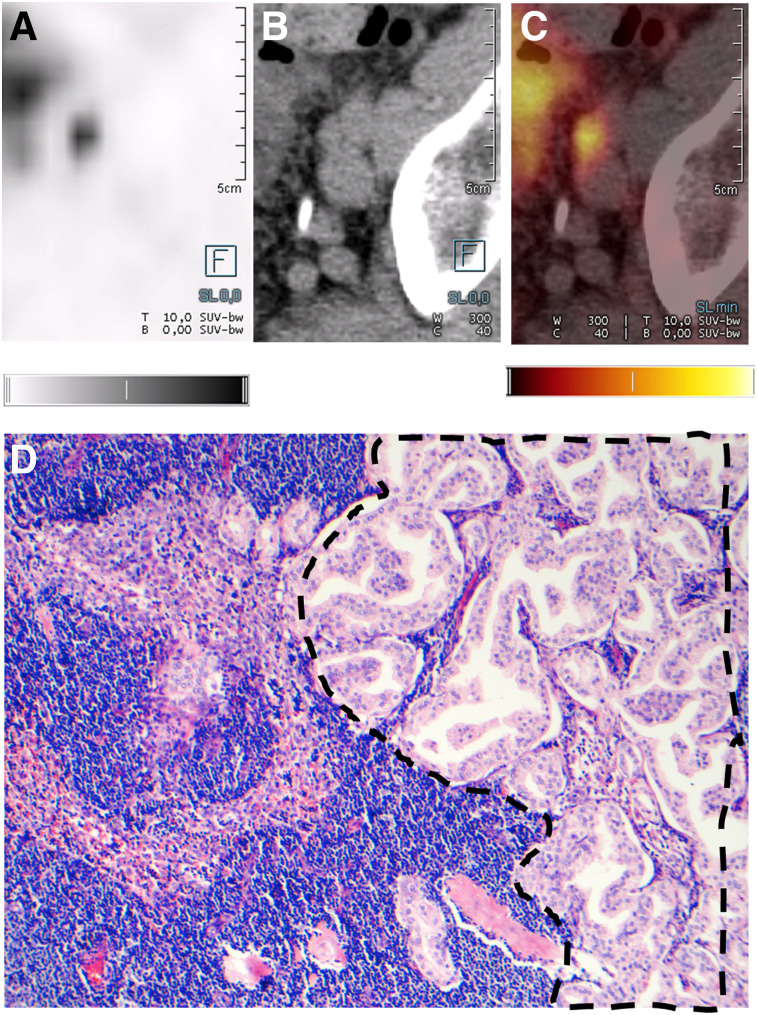
(A–C) Axial ^18^F-PSMA-1007 PET (A), CT (B), and ^18^F-PSMA-1007 PET/CT (C) images of 67-year old patient with prostate adenocarcinoma (prostate-specific antigen level, 21.0 ng/mL; Gleason score, 3 + 4; T3bN1M0). (D) Confirmed histopathologic staining (hematoxylin and eosin, ×10) of acinar structures of prostate adenocarcinoma (dashed outline) adjacent to normal LN structures. Quantitative gray scale and color scale represent SUV from 0.00 to 5.00.

**FIGURE 3.**
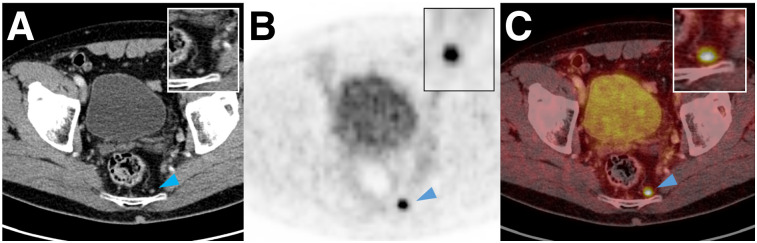
Axial CT (A), ^18^F-PSMA-1007 PET (B), and ^18^F-PSMA-1007 PET/CT (C) images of 65-y-old patient with prostate adenocarcinoma (initial PSA, 23.8 ng/mL). There is 1 positive LN (arrowhead) close to plexus sacralis that was not removed during surgery. Patient’s prostate-specific antigen level continued to rise after surgery, indicating that retained node was malignant.

**FIGURE 4.**
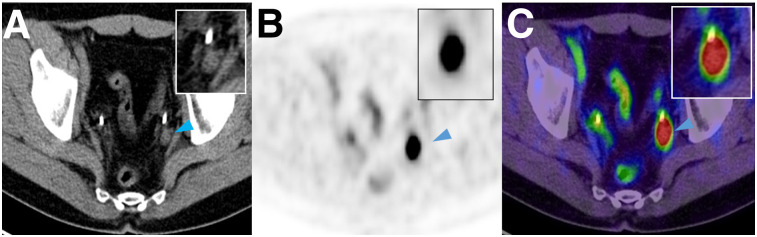
Axial CT (A), ^18^F-PSMA-1007 PET (B), and ^18^F-PSMA-1007 PET/CT (C) images of suspected lesion (arrowhead) in left iliac chain with SUV of 20.8. Histopathology revealed LN conglomerate of 3 positive LNs.

Overall sensitivity, specificity, PPV, and NPV on lesion-based analysis were 71.2%, 99.5%, 91.3%, and 97.9%, respectively (Table 2). In total, 104 of 1,746 (6.0%) resected LNs were larger than 3 mm and positive on histopathology, and of these 104 LNs, ^18^F-PSMA-1007 PET/CT correctly detected 85 (81.7%). For LNs larger than 3 mm, sensitivity, specificity, PPV, and NPV on lesion-based analysis were 81.7%, 99.6%, 92.4%, and 98.9%, respectively (Table 3). Cases of correctly staged patients are shown in Figures 1 and 2. Cases of mismatch between imaging and histopathology are shown in Figures 3 and 4.

**TABLE 2:** Lesion-Based Analysis of PET Findings and Histopathology for All Malignant Nodes

	LN histopathology		
LN ^18^F-PSMA-1007-PET/CT	Positive	Negative	Sums
Positive	True-positive (A) = 84	False-positive (B) = 8	A + B = 92
Negative	False-negative (C) = 34	True-negative (D) = 1,620	C + D = 1,654
Sums	A + C = 118	B + D = 1,628	N = 1,746

**TABLE 3:** Lesion-Based Analysis of PET Findings and Histopathology for Nodes Larger Than 3 mm in Histopathology

	LN histopathology		
LN ^18^F-PSMA-1007-PET/CT	Positive	Negative	Sums
Positive	True-positive (A) = 85	False-positive (B) = 7	A + B = 92
Negative	False-negative (C) = 19	True-negative (D) = 1,635	C + D = 1,654
Sums	A + C = 104	B + D = 1,642	N = 1,746

### Patient-Based Analysis

A patient-based analysis was performed (Supplemental Tables 2 and 3). The fraction of detected positive and negative nodes per patient was calculated. Of the 96 patients in the study, 31 (32.3%) had proven LN metastasis based on a histopathologic size larger than 3 mm (N+); 65 patients were categorized as N0.

The patient-based analysis showed a sensitivity of 85.9% (SE, 4.38%) for detecting LNs larger than 3 mm and a specificity of 99.5% (SE, 0.25%). The patient-based sensitivity for detecting LNs regardless of size was 73.5% (SE, 5.82%), and the specificity was 99.4% (SE, 0.28%).

Moreover, a rather strict analysis was performed for agreement between histopathology and PET/CT with regard to the exact number of positive nodes. True-positives and true-negatives were defined as the exact consensus for the number of positive and negative nodes. A false-positive was noted if PET/CT detected more LNs than histopathology. A false-negative was counted when there were more positive nodes on histopathology. Results for LNs larger than 3 mm for this strict analysis were as follows: sensitivity, 64.3% (SE, 11.29%); specificity, 91.2% (SE, 3.60%); PPV, 75%; and NPV, 86.1%. For all LNs regardless of size, sensitivity was 50% (SE, 13.36%), specificity was 89.7% (SE, 3.89%), PPV was 66.7%, and NPV was 81.3%.

### Diagnostic Accuracy in Biochemical Recurrence

Nine patients with biochemical recurrence were included. In total, 143 LNs were resected from those patients. For LNs larger than 3 mm, the correlation between imaging and histopathology showed a sensitivity of 78.6%, a specificity of 100%, a PPV of 100%, and a NPV of 97.7% in a lesion-based analysis. When all lesions regardless of size were included, sensitivity was 57.9%, specificity 100%, PPV 100%, and NPV 93.9%.

## DISCUSSION

Staging and restaging of prostate cancer are essential to determine the most appropriate treatment modality. It is vital that any imaging test used for staging be reliable to avoid any unnecessary or harmful intervention. This study demonstrated that the tracer ^18^F-PSMA-1007 has high sensitivity and extraordinary specificity for LN metastases in prostate cancer patients undergoing lymphadenectomy.

Table 4 gives an overview of the detected rates for ^68^Ga-PSMA-11 and ^18^F-PSMA-1007. These rates equal or are even higher than those in comparative reports. Of note, specificity was nearly perfect and PPV very high, indicating that false-positives are rare.

**TABLE 4:** Results After Comparison Between PET/CT and Histopathology

Tracer	Study	Number of patients	Sensitivity	Specificity	PPV	NPV
^68^Ga-PSMA-11	Hope, 2019 (17)	266 patients, 29 articles	74%	96%	93%	85%
	Perera, 2016 (4)	239 patients, 5 studies	80%	97%	—	—
	Afshar-Oromieh, 2015 (3)	42 patients	76.6%	100%	100%	91.4%
	Kuten, 2020 (18)	16 patients	85.7%	98.2%	96.8%	91.5%
^18^F-PSMA-1007	Kuten, 2020 (18)	16 patients	100%	90.9%	87.5%	100%
	Giesel, 2017 (8)	8 patients	94.7%	—	—	—
	Kesch, 2017 (9)	10 patients	71%	81%	83%	68%
	Sprute et al. (this work)	96 patients, overall	71.2%	99.5%	91.3%	97.9%
		96 patients, lymph nodes >3mm	81.7%	99.6%	92.4%	98.9%

In a prospective evaluation, the diagnostic accuracy of ^68^Ga-PSMA for N-staging in the setting of biochemical recurrence after radical prostatectomy was evaluated. The sensitivity of ^68^Ga-PSMA-11 PET/MRI was 72%–100% and the specificity 96%–100% in a regional analysis (12). In a systematic review and metaanalysis by Kimura et al. (13), 14 studies were included, looking at the performance of ^68^Ga-PSMA-11 PET/CT in patients with biochemical recurrence. The lesion-based sensitivity was 84% and the specificity 97%. Those results are comparable to the numbers published in this study for biochemical recurrence.

Correlating imaging and histopathologic findings in LNs is challenging. To accurately account for nodes, the imaging must be reviewed with the surgeon since it is technically not possible to remove every node safely. Positive PET/CT findings outside the surgical area (e.g., perilous location close to other organs or vessels) were excluded from the evaluation. Moreover, there may be difficulties colocalizing imaging findings to specific histologic findings. A typical example of such difficulties is shown in Figure 3.

In 2 patients, ^18^F-PSMA-1007 PET/CT detected lesions with extremely high SUVs. In these cases, histopathology demonstrated the confluence of 2 or more nodes directly adjacent to each other. Therefore, imaging showed a LN conglomerate and not a single suggestive node (Fig.4).

Other limitations of this study are the retrospective design, with an inherent selection bias; the lack of standardization of the imaging protocols among the different centers; and a long interval between imaging and surgery.

Additionally, some nodes contained only small amounts of cancer, likely below the detection threshold of PET/CT; therefore, 2 approaches were used. In the first, all nodes positive on histopathology were considered. In the second, only LN metastases larger than 3 mm were considered.

In a study of LN size, lesions between 2 and 3 mm were detected with a sensitivity of only 13.3%, whereas those measuring 5–6 mm were seen in 57.4% of the cases (14). Maurer et al. revealed that most of the LNs that were missed on ^68^Ga-PSMA PET/CT measured 3 ± 1 mm (15). In a recent study, the median size of the LNs that were not detected by^18^F-radiohybrid-PSMA PET was 4.5 mm (range, 0.3–15 mm) (16).

## CONCLUSION

^18^F-PSMA-1007 PET/CT demonstrates a high sensitivity and extraordinary specificity in detecting affected LNs in prostate cancer. The results are at least comparable to existing data for ^68^Ga-labeled PSMA tracers on a per-lesion basis.

## DISCLOSURE

A patent application has been filed for PSMA-1007 by Uwe Haberkorn and Frederik Giesel. Frederik Giesel is medical advisor for ABX advanced biochemical compound, Telix Pharmaceuticals and Sofie Biosciences. Matthias Eiber received research and travel support from ABX and Blue Earth Diagnostics and holds patent rights on rhPSMA. Kambiz Rhabar has received consultant fees from Bayer and ABX and lectureship fees from Janssen Cilag, Amgen, AAA and SIRTEX, and travel expenses from Endocyte as unpaid member of the VISION Trial Steering Committee. Dr. Juergen Debus reports grants from Viewray Inc., grants from CRI The Clinical Research Institute GmbH, grants from Accuray International Sari, grants from RaySearch Laboratories AB, grants from Vision RT Limited, grants from Merck Serono GmbH, grants from Astellas Pharma GmbH, grants from Astra Zeneca GmbH, grants from Siemens Healthcare GmbH, grants from Solution Akademie GmbH, grants from Egomed PLC Surrey Research Park, grants from Quintiles GmbH, grants from Pharmaceutical Research Associates GmbH, grants from Boehringer Ingelheim Pharma GmbH&CoKG, grants from PTW-Freiburg Dr. Pychlau GmbH, and grants from Nanobiotix S.A, outside the submitted work. Travel support was provided from ABX. No other potential conflict of interest relevant to this article was reported.

**KEY POINTS**

**QUESTION:** Can ^18^F-PSMA-1007 PET/CT reliably detect malignant LNs?
**PERTINENT FINDINGS:** In this investigation assembling over 1,700 LNs in 96 patients from 5 centers around the globe, ^18^F-PSMA-1007 PET/CT demonstrated a high sensitivity and extraordinary specificity in detecting affected LNs in prostate cancer.
**IMPLICATIONS FOR PATIENT CARE:** ^18^F-PSMA-1007 PET/CT can be used for staging and restaging in prostate cancer and reliably detects positive LNs.
